# Therapeutic potential of Xihuang Pill in colorectal cancer: Metabolomic and microbiome-driven approaches

**DOI:** 10.3389/fphar.2024.1402448

**Published:** 2024-12-02

**Authors:** Chen Zhang, Conglu Sui, Xiaona Ma, Chongyang Ma, Xinhui Sun, Changming Zhai, Peng Cao, Yue Zhang, Jinjun Cheng, Tong Li, Jiayang Sai

**Affiliations:** ^1^ Department of oncology, The Third Affiliated Hospital, Beijing University of Chinese Medicine, Beijing, China; ^2^ School of Traditional Chinese Medicine, Capital Medical University, Beijing, China; ^3^ School of Life Sciences, Beijing University of Chinese Medicine, Beijing, China; ^4^ National Institute of TCM Constitution and Preventive Medicine, Beijing University of Chinese Medicine, Beijing, China

**Keywords:** colorectal cacner, Xihuang Pill, 5-fu (5-fluorouracil), untarget metabolomics, gut micobiota

## Abstract

**Introduction:**

The Xihuang Pill (XHP), a venerated traditional Chinese medicine, has demonstrated significant anti-cancer capabilities. Despite its proven efficacy, the scarcity of comprehensive pharmacological studies limits the widespread application of XHP. This research endeavor seeks to demystify the therapeutic underpinnings of XHP, particularly in the realm of colorectal cancer (CRC) therapy.

**Methods:**

In this study, mice harboring CT26 tumors were divided into four groups, each administered with either XHP monotherapy, 5-fluorouracil (5-FU), or a combination of both. The tumor growth trajectory was closely monitored to evaluate the effectiveness of these anti-neoplastic interventions. Advanced techniques, including 16S-rDNA gene sequencing and ultra-high performance liquid chromatography-tandem mass spectrometry (UHPLC-MS/MS), were harnessed to scrutinize the gut microbiota and serum metabolite profiles. Immunohistochemical assays were employed to gauge the expression levels of CD4, CD8, and Foxp3, thereby providing insights into the dynamics of tumor-infiltrating lymphocytes within the tumor microenvironment.

**Results:**

Our findings indicate that XHP effectively suppresses the initiation and progression of colorectal tumors. The combinatorial therapy of XHP with 5-FU exhibited an enhanced inhibitory effect on tumor growth. Metabolic profiling revealed that XHP induced notable metabolic shifts, particularly impacting pathways such as steroid hormone synthesis, arachidonic acid metabolism, purine biosynthesis, and renin secretion. Notably, 17α-ethinyl estradiol and α-ergocryptine were identified as serum metabolites with the most substantial increase following XHP administration. Analysis of the gut microbiome suggested that XHP promoted the expansion of specific bacterial taxa, including *Lachnospiraceae_NK4A136_group*, *Clostridiales*, *Desulfovibrionaceae*, and *Anaerotignum_sp*., while suppressing the proliferation of others such as *Ligilactobacilus*, *Lactobacillus_taiwanensis*, and *Candidatus_saccharimonas*. Immunohistochemical staining indicated an upregulation of CD4 and CD8 post-XHP treatment.

**Conclusion:**

This study delineates a potential mechanism by which XHP inhibits CRC tumorigenesis through modulating the gut microbiota, serum metabolites, and reshaping the tumor immune microenvironment in a murine CRC model. These findings contribute to a more profound understanding and potentially broaden the clinical utility of XHP in oncology.

## Graphical Abstract

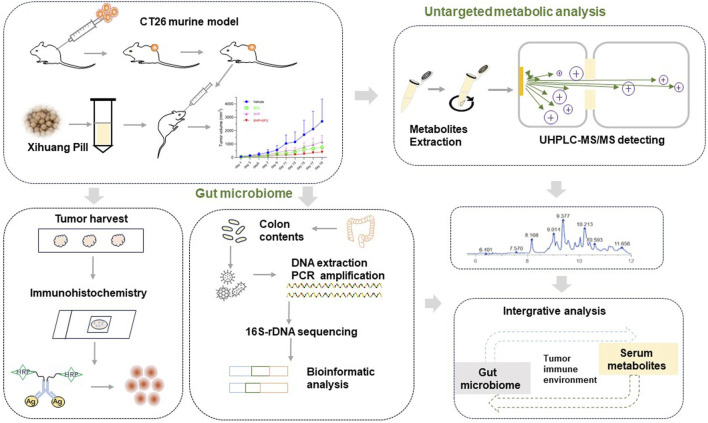

## Introduction

Colorectal cancer (CRC) stands as the third most frequently diagnosed malignant tumor globally (Basak et al., 2020; Lotfollahzadeh et al., 2023), with the United States and China witnessing the fourth and fifth highest incidence rates, respectively (Miller et al., 2019; Siegel et al., 2020; Wu et al., 2019). Remarkably, the prevalence of CRC is on the rise, largely attributed to the evolution of dietary habits over recent years (Siegel et al., 2013; Yang et al., 2010). Chemotherapy continues to underpin the primary treatment strategy for CRC, with combination therapies often recommended to enhance treatment outcomes. The advent of immune checkpoint blockade (ICB) therapy once sparked considerable optimism; however, its clinical efficacy in CRC has not matched the success observed in lung cancer or melanoma (Diaz and Le, 2015; Topalian et al., 2012; Yu et al., 2019). Subsequent investigations have indicated that CRC patients with high microsatellite instability (MSI-H) or deficient mismatch repair (dMMR) exhibit heightened sensitivity to ICB therapy, in contrast to those with microsatellite stable (MSS) tumors who show minimal responsiveness to immunotherapy (Du et al., 2022). Given that only a small fraction (approximately 15%) of CRC patients presents with MSI-H, with the majority, 85%, exhibiting MSS (Papadopoulos, 1999; Vilar and Tabernero, 2013), it is imperative to enhance clinical responses and survival rates for the prevalent MSS-CRC patient population.

The Xihuang Pill (XHP), esteemed within the Traditional Chinese Medicine (TCM) repertoire, boasts a legacy that dates back multiple centuries (Li et al., 2020; Su et al., 2018). Historically, XHP has served not only as a complementary therapy but also as a primary alternative in the treatment of a range of malignancies, with a particular focus on cancers of the breast, lung, and colon (Cao et al., 2022). Contemporary research underscores the possibility that XHP could significantly boost the potency of chemotherapeutic interventions and precision cancer treatments (Cao et al., 2022; Fu et al., 2020). While XHP has demonstrated efficacy in clinical settings for the treatment of CRC, its full medicinal potential in this domain has not yet been fully realized. The extent of its therapeutic capabilities in addressing CRC remains largely untapped, awaiting a deeper exploration of its multifaceted healing properties.

TCM has recently been shown to exert disease-modifying effects through interactions with the gut microbiota. Metabolites generated by intestinal flora can impact host pathology by entering the bloodstream, offering a new perspective on how TCM may influence various conditions (Jiao et al., 2023). Given that XHP contains compounds in trace amounts that are poorly absorbed, investigating its interaction with the gut microbiota could uncover the underlying mechanisms responsible for its systemic influence. Although it is acknowledged that metabolic imbalances might arise without direct involvement of gut microbiota disruptions, adopting a microbiome-metabolomics strategy to scrutinize XHP could unveil innovative perspectives. Our investigative journey led us to a fascinating revelation: estrogen, a hormone predominantly associated with female physiology, may exert a crucial influence on XHP’s anti-CRC efficacy. This discovery opens new avenues for understanding the intricate relationship between hormonal activity and the therapeutic effects of traditional medicines like XHP in the context of CRC treatment.

## Materials and methods

### Preparation of XHP solution

XHP, incorporated into the Chinese Pharmacopoeia, is formulated with a blend of botanical drugs including *Calculus Bovis* (gallstones of *Bos Taurus domesticus Gmelin*), *Moschus* (secretion of *Moschus berezovskii*), *Olibanum* (resin of *Boswellia carterii Birdw.*), *Myrrha* (resin from *Commiphora myrrha Engl.* Or *Commiphora molmol Engl.*). XHP was purchased from Tianjin Tianshili (Liaoning) Pharmaceutical Co., Ltd. (Batch number: 20140726). It comes in a standard packaging of 0.1 g per pill, with a total of 30 bottles per box. The formulation of the pills is meticulously balanced, with a composition ratio of *Moschus*, *Calculus Bovis*, *Myrrha*, and *Olibanum* at 15:15:550:550, respectively. The pills were crushed into a fine powder and reconstituted in sterile distilled water to prepare a stock solution with a concentration of 78 mg/mL. This stock solution was then stored at −4°C to preserve its integrity.

Preparation of XHP solution required for ultra-high performance liquid chromatography-tandem mass spectrometry (UHPLC-MS/MS): 1 g of XHP powder was weighed, and combined with 20 mL methanol. The mixture was sonicated in an ice bath for 20 min, extracted twice, and centrifuged at 4,000 r/min for 5 min. After that, the supernatant was vacuum concentrated and reconstituted to a 10 mL volume with 80% methanol. Finally, the mixture was filtered through a 0.22 μm membrane.

The UHPLC-MS/MS analysis was conducted using a Hypersil Gold column (100 × 2.1 mm, 1.9 μm). A 12 min linear gradient elution system was employed for the separation process. Sample injections were performed at a flow rate of 0.2 mL/min. In the positive ion mode, the mobile phases consisted of eluent A (0.1% formic acid in water, v/v) and eluent B (methanol). For the negative ion mode, the mobile phase A was a 5 mM solution of ammonium acetate adjusted to pH 9.0, with phase B being methanol. The mass spectrometry parameters were optimized as follows: spray voltage set to 3.5 kV, capillary temperature maintained at 320°C, sheath gas flow rate at 35 psi, auxiliary gas flow rate at 10 L/min, S-lens RF level at 60, and auxiliary gas heater temperature at 350°C.

### Animals

Female BALB/c mice, aged 6 weeks, were procured from Vital River Laboratory Animal Technology Co., Ltd. (Beijing, China). The animals were housed in a pathogen-free facility under conditions of constant temperature and humidity. Ethical approval for the experimental protocol was granted by the Committee for Animal Experimentation of Beijing University of Chinese Medicine (Beijing, China), in accordance with the approval number BUCM-2023022302-1100.

### Cell cultures

The murine colon carcinoma cell line CT26 (1101MOU-PUMC000275) was obtained from the National Infrastructure of Cell Line Resource. The cells were cultured in Roswell Park Memorial Institute (RPMI)-1,640 medium supplemented with 10% fetal bovine serum and 1% penicillin/streptomycin, procured from Beijing Aoqing Biotechnology Co., Ltd., China.

### Grouping and drug administration

All experimental animals were adaptively nourished for a 1 week period under stringent specific pathogen-free (SPF) conditions, with both food and water supplied without restriction. Then CT26 cells were subcutaneously injected into the right rear flank region of BALB/c mice (6 mm superior to the hind limbs) at a density of 5×10^5^ cells per site in a 100 µL volume. By the third day post-injection, palpable tumors, approximately 5 mm in size, had developed. Subsequently, the mice were randomly assigned into four treatment cohorts: (1) a vehicle control group, (2) a 5-FU treatment group receiving 50 mg/kg via intraperitoneal injection once weekly, (3) a XHP treatment group administered 0.78 g/kg orally daily, and (4) a co-therapy group receiving a combination of 5-FU (50 mg/kg, intraperitoneal, once weekly) and XHP (0.78 g/kg, oral, daily).

**FIGURE 1:**
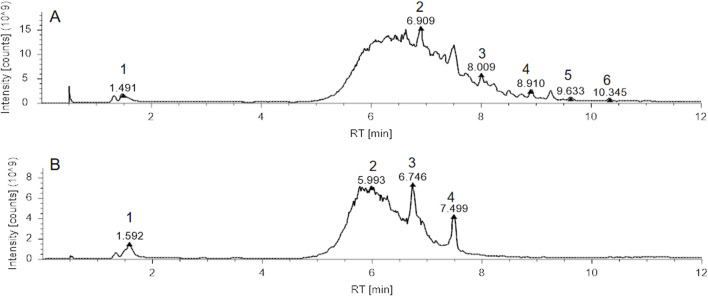
Total Ion Chromatogram (TIC) analysis of Xihuang Pill (XHP). **(A)** TIC of XHP in positive ion mode, with identified compounds: 1. Betaine, 2. Tetrahydrocortisone, 3. Dehydrotumulosic acid, 4. D-(+)-Camphor, 5. Ursolic acid, 6. Acetyl-11-keto-β-boswellic acid. **(B)** TIC of XHP in negative ion mode, with identified compounds: 1. Maltotetrose, 2. Azelaic acid, 3. ST 24:1; O4; T, 4. Cholic acid. Abbreviations: XHP, Xihuang Pill; RT, Retention Time..

The dosages of 5-FU and XHP were selected in accordance with previous studies (Li et al., 2020; Li et al., 2022). The administered dose of XHP to the mice was calibrated to reflect the clinical equivalent dose of 6 g/day. Tumor dimensions were measured every 2 days using a caliper, and tumor volume (Tv) was calculated employing the formula: Tv = (length× width^2^)/2 (Yu et al., 2019). Upon completion of the 3 week treatment period, mice underwent anesthesia induced by intraperitoneal injection of sodium pentobarbital at a dosage of 100 mg/kg. Subsequently, blood was harvested via cardiac puncture, followed by centrifugation at 4,000 rpm for 10 min at 4°C to obtain serum samples. The tumors were then excised, weighed, and prepared for subsequent analyses. A schematic representation of the experimental design is presented in Figure 1.

### Metabolites extraction and analysis

Chromatographic analysis of serum samples was conducted utilizing a Vanquish UHPLC system (Thermo Fisher, Germany) interfaced with an Orbitrap Q ExactiveTM HF mass spectrometer. Initially, serum samples were thawed, and to 100 µL of serum, 400 µL of an 80% methanol solution was added. The mixture was then incubated on ice for 5 min prior to centrifugation at 15,000 g for 20 min at 4°C. The supernatant was subsequently diluted with LC-MS grade water to achieve a final methanol concentration of 53%. The diluted solution was transferred to a new Eppendorf tube and re-centrifuged under the same conditions to ensure clarity and purity.

Serum metabolomic profiling was conducted using identical conditions as those previously established for the XHP study. Raw data acquired from the UHPLC-MS/MS experiments were processed for peak alignment and quantitation using Compound Discoverer 3.3 software (Thermo Fisher Scientific) (Yu et al., 2023). Following normalization to total ion intensity, metabolite peaks were correlated to libraries on mzCloud database (https://www.mzcloud.org/), mzVault, and MassList for precise identification and relative quantitation. Subsequently, metabolite identification was refined by analyzing their fragmentation patterns against the KEGG database, the Human Metabolome Database, and the LIPID Maps database.

To discern variations across experimental groups, we conducted Principal Component Analysis (PCA) and Orthogonal Partial Least Squares Discriminant Analysis (OPLS-DA) model utilizing the metaX software suite (Li et al., 2023). The Variable Importance in Projection (VIP) scores and fold change (FC) values were computed to identify key metabolites, following the methodology described by Jansson et al. (Jansson et al., 2009). Metabolites were classified as differentially abundant if they exhibited a VIP score greater than 1, a *p*-value below 0.05, and a FC of 2 or greater, or 0.667 or less, as per the criteria established by Haspel et al., and Sreekumar et al. (Haspel et al., 2014; Sreekumar et al., 2009). To visually represent these differential metabolites, particularly in comparisons between the vehicle and XHP groups, we generated a volcano plot and a heat map.

### 16S-rDNA gene sequencing and bioinformatic analysis

#### DNA extractions

Microbial DNA from the mouse gut was extracted utilizing the cetyltrimethylammonium bromide (CTAB) method, following the protocol provided by the manufacturer (Real Times Biotechnology co. Ltd, Beijing, China, catalog number RTG2405-01). Post-extraction, DNA concentration and purity were assessed, and the samples were subsequently diluted to a working concentration of 1 ng/μL. Aliquoted DNA was stored at −80°C for subsequent PCR analysis. For 16S-rDNA amplification, primers with unique 5′barcodes and universal sequencing primers were employed (Supplementary Table S1). PCR amplification was performed, and the success of amplification was verified using 2% agarose gel electrophoresis. Amplicons were purified using AMPure XT beads (Beckman Coulter Genomics, Danvers, MA, United States) to remove excess primers and primer-dimers, ensuring clean sequencing templates. Purified products were quantified using the Qubit fluorometer (Invitrogen, United States) to ensure accurate library preparation. The qualified sequencing libraries are denatured with NaOH to form single strands, and sequenced using a NovaSeq 6,000 sequencer with 2 × 250 bp paired-end reads (NovaSeq 6000 SP Reagent Kit, 500 cycles, United States). The data containing sequencing numbers fewer than 100 base pairs were excluded.

#### Bioinformatic analysis

Raw sequencing reads of the 16S-rDNA gene were processed utilizing UPARSE software, version 7.1. Operational taxonomic units (OTUs) were clustered at a 97% similarity threshold, a common practice in microbial community analysis. The richness and evenness of the microbial communities were assessed through the calculation of α-diversity indices, including Chao1 and Shannon, performed with R software (version 3.4.4). To evaluate the community structure and compositional differences among samples, β-diversity was estimated using QIIME2 (version 2019.7). The resulting data were visualized through Principal Coordinate Analysis (PCoA) and Partial Least Squares Discriminant Analysis (PLS-DA), conducted with the R vegan and mixOmics packages, respectively. The significance of the observed clustering patterns was determined using Permutational Analysis of Variance (PERMANOVA). The identification of a characteristic microbiome was achieved through the Linear Discriminant Analysis Effect Size (LEfSe) method, which helps to reveal the biologically relevant features of the microbial composition. Correlations between the gut microbiota and serum metabolites were explored with R language (version 3.4.4), and the results were visualized in a heatmap generated by the OmicStudio tools (https://www.omicstudio.cn).

#### Immunohistochemical staining

Tissue specimens were fixed, dehydrated, and embedded in paraffin for sectioning. Using a microtome, 5 µm sections were cut and mounted on positively charged glass slides. Slides were deparaffinized and rehydrated pre-immunostaining. Antigen retrieval was performed followed by incubation at 4°C with primary antibody: Ki67 (catalog number GB121141-100), CD8 (catalog number GB15068-100), CD4 (catalog number GB300601-M-10 μg), and FoxP3 (catalog number GB112325-100), all diluted 1:500 in diluent within a humid chamber. After primary antibody incubation, slides were treated with biotinylated secondary antibodies and detected using a streptavidin-HRP complex for antigen-antibody interaction visualization. All antibodies were procured from Servicebio (Wuhan, China). The immunostained slides were examined and imaged using the Caseviewer 2.4 system. The integral optical density (IOD) of positive staining was quantitatively measured using Image Pro Plus 6.0 (MediaCybernetics) and adjusted for the area of staining (Fiore et al., 2012).

#### Statistical analysis

Data are reported as the mean value ±standard deviation (SD) to illustrate the central tendency and dispersion of the measurements. Statistical analyses were conducted using GraphPad Prism software, version 9. Comparisons among multiple groups were evaluated using one-way analysis of variance (ANOVA), followed by *post hoc* tests when appropriate. For pairwise comparisons, a two-tailed Student’s t-test was applied to determine the statistical significance of differences between two groups. A *p*-value of less than 0.05 was set as the threshold for statistical significance.

## Results

### LC/MS analysis of XHP

Upon completion of the LC/MS analysis, a qualitative examination of the compounds within XHP was executed. The resulting chromatographic peaks are illustrated in Figure 1. This analytical endeavor successfully identified distinct compounds within the XHP formulation. Prominent among these were betaine, tetrahydrocortisone, dehydrotumulosic acid, d-(+)-camphor, ursolic acid, acetyl-11-keto-β-boswellic acid, maltotetraose, azelaic acid, and cholic acid, each contributing to the complex pharmacological profile of XHP.

### Anti-tumor effects of XHP

**FIGURE 2:**
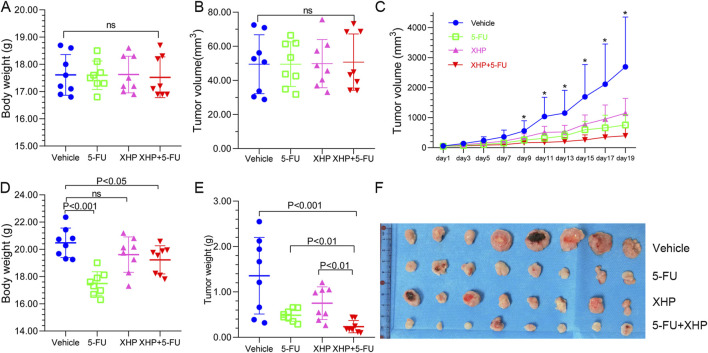
Xihuang Pill (XHP) inhibits colorectal tumorigenesis in the CT26 murine model. **(A)** Pre-treatment body weight of mice across four groups (n = 8). **(B)** Initial tumor volume in the four groups (n = 8). **(C)** Tumor growth trajectories of the four groups (n = 8). **(D)** Final body weight of mice in the four groups at the experiment endpoint (n = 8). **(E)** Final tumor weight in the four groups at the experiment endpoint (n = 8). **(F)** Tumors harvested from the four groups at the experiment endpoint (n = 8). * Represents *p* < 0.05.

The CT26 tumor-bearing mouse model was employed to evaluate the anti-tumor efficacy of XHP. As depicted in Figures 2A, B, initial tumor volumes and mouse weights were comparable across all groups, indicating equivalent baseline conditions. After 3 weeks of treatment, significant variations in both mouse weight and tumor volume were observed (Figures 2C–F). Mice in the vehicle, XHP, and combination therapy groups exhibited considerable weight gain, whereas those treated with 5-FU alone showed minimal weight change. Moreover, compared to the vehicle group, treatments with 5-FU, XHP, and the combination therapy all resulted in a reduction of tumor size. Notably, the combination therapy group demonstrated the most pronounced tumor growth inhibition. Although 5-FU alone had a more pronounced inhibitory effect on tumor growth than XHP, the combined treatment was superior in terms of tumor reduction. These findings suggest that XHP possesses anti-tumor properties against CRC, and its combination with 5-FU elicits a synergistic anti-tumor effect.

### Metabolomics analysis

**FIGURE 3:**
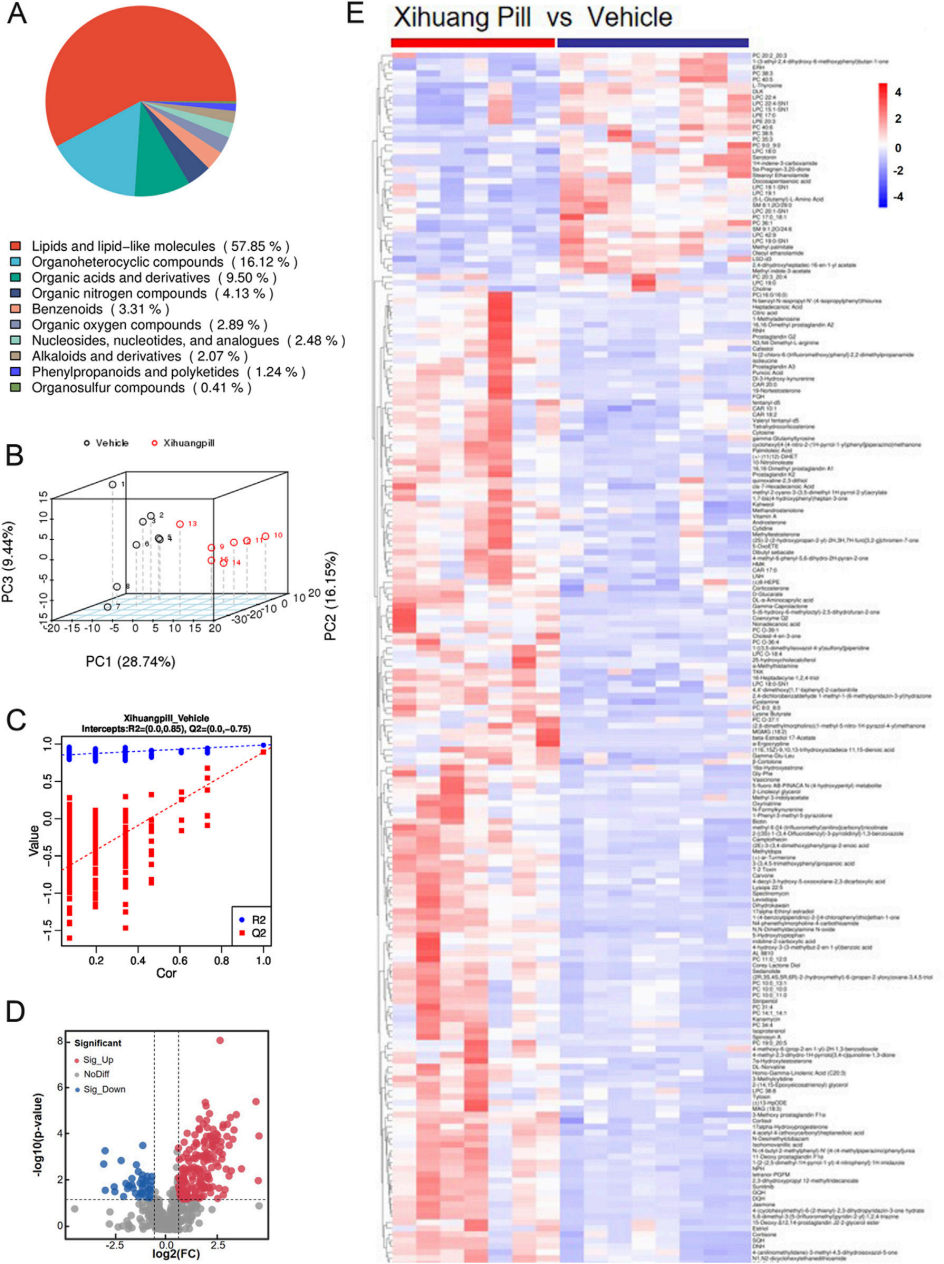
Metabolomic profiling of vehicle and Xihuang Pill (XHP) groups via ultra-high performance liquid chromatography-tandem mass spectrometry (UHPLC-MS/MS) positive electrospray ionization (ESI+) Mode. **(A)** A pie chart depicting the classification of metabolites. **(B)** Principal Component Analysis (PCA) score plots illustrating the distribution of metabolomic data. **(C)** Orthogonal Partial Least Squares-Discriminant Analysis (OPLS-DA) score plots showcasing the metabolite discrimination between groups. **(D)** Volcano plots highlighting the differential metabolites between the vehicle and XHP groups. **(E)** A heatmap representing the differential metabolites between the vehicle and XHP groups.

**FIGURE 4:**
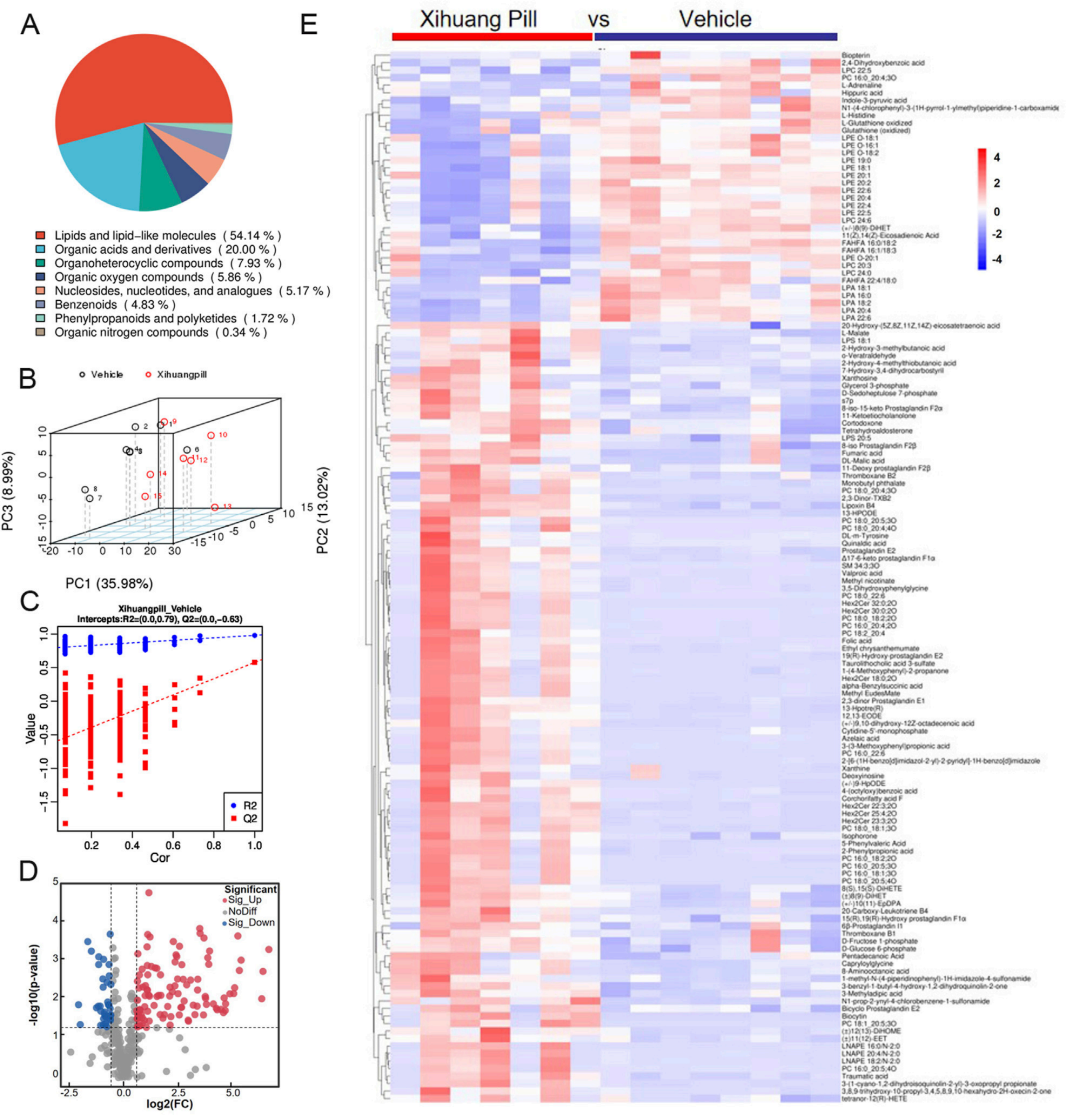
Metabolomic profiling of vehicle and Xihuang Pill (XHP) groups via ultra-high performance liquid chromatography-tandem mass spectrometry (UHPLC-MS/MS) negative electrospray ionization (ESI-) Mode. **(A)** A pie chart depicting the classification of metabolites. **(B)** Principal Component Analysis (PCA) score plots illustrating the distribution of metabolomic data. **(C)** Orthogonal Partial Least Squares-Discriminant Analysis (OPLS-DA) score plots showcasing the metabolite discrimination between groups. **(D)** Volcano plots highlighting the differential metabolites between the vehicle and XHP groups. **(E)** A heatmap representing the differential metabolites between the vehicle and XHP groups.

Untargeted metabolite profiling utilizing UHPLC-MS/MS was conducted on serum samples obtained from mice in the vehicle and XHP treatment groups to elucidate the endogenous metabolic responses induced by XHP administration. After the exclusion of hemolytic samples, a total of seven samples from the XHP group and eight from the vehicle group were subjected to metabolomic analysis. The metabolites identified through UHPLC-MS/MS analysis were systematically classified based on their chemical properties. Lipids and their analogous molecules were notably abundant, constituting a significant majority of the metabolites detected in both positive (ESI+) and negative ion modes (ESI-). Specifically, they represented 57.85% in ESI+ and 54.14% in ESI-. Organoheterocyclic compounds were also prominently detected, contributing to 16.12% of the metabolites in ESI+ and 7.93% in ESI-. Organic acids and their derivatives were another significant group, with their presence being 9.5% in ESI+ and 20% in ESI-. Organic nitrogen compounds were less prevalent, comprising a mere 4.13% in ESI+ and an even smaller fraction of 0.34% in ESI-. Benzenoid compounds were identified as well, contributing to 3.31% in ESI+ and 4.83% in ESI-. Lastly, organic oxygen compounds were detected at lower levels, representing 2.89% in ESI+ and 5.86% in ESI- (as illustrated in Figures 3A, 4A).

An unsupervised multivariate analysis was conducted using PCA to discern the metabolic variance between the two groups. The PCA plot, as depicted in Figures 3B, 4B, illustrates a distinct separation between the groups, indicating a significant metabolic divergence. All samples are observed to fall within the 95% confidence ellipse, signifying the robustness of the PCA model. In the ESI+, the first three principal components (PC1, PC2, and PC3) explained the variance of 28.74%, 16.15%, and 9.44%, respectively. In the ESI-, the corresponding percentages were 35.98%, 13.02%, and 8.99%. These contributions to the total variance underscore the model’s capacity to differentiate the metabolic profiles of the groups. Subsequently, an OPLS-DA model was established, yielding satisfactory R2 and Q2 values of 0.85 and 0.75 in ESI+, and 0.79 and 0.63 in ESI-, respectively (Figures 3C, 4C). These values reflect the model’s excellent fit and predictive ability. The pronounced differences in metabolite profiles between the groups suggest distinct biological responses to XHP treatment.

### Detection and identification of metabolites

Applying a stringent threshold of VIP greater than 1.0, FC exceeding 1.5 or less than 0.667, and a *p*-value below 0.05, our metabolomic analysis successfully identified a total of 203 metabolites in the ESI+ and 140 metabolites in the ESI-. In ESI+, 164 metabolites exhibited upregulation, while 39 were downregulated. Conversely, in ESI-, the numbers were 104 and 38, respectively, for upregulated and downregulated metabolites.

The volcano plots presented in Figures 3D, 4D provide a visual representation of the metabolites identified under the specified thresholds. In the ESI+, lipids and lipid-like molecules emerged as the most prevalent, with 48 upregulated metabolites and 15 downregulated metabolites constituting 29.26% and 38.46% of the total differential metabolites, respectively. Notably, within the lipid class, fatty acids and their derivatives, as well as steroids and steroid-related compounds, were predominant, representing 45.83% and 31.25% of the upregulated lipids, respectively. In the ESI-, 34 metabolites were found to be upregulated, of which 18 (50%) were lipids and lipid-like molecules. These findings underscore the significant alterations in lipid metabolism in response to the treatment conditions.

**FIGURE 5:**
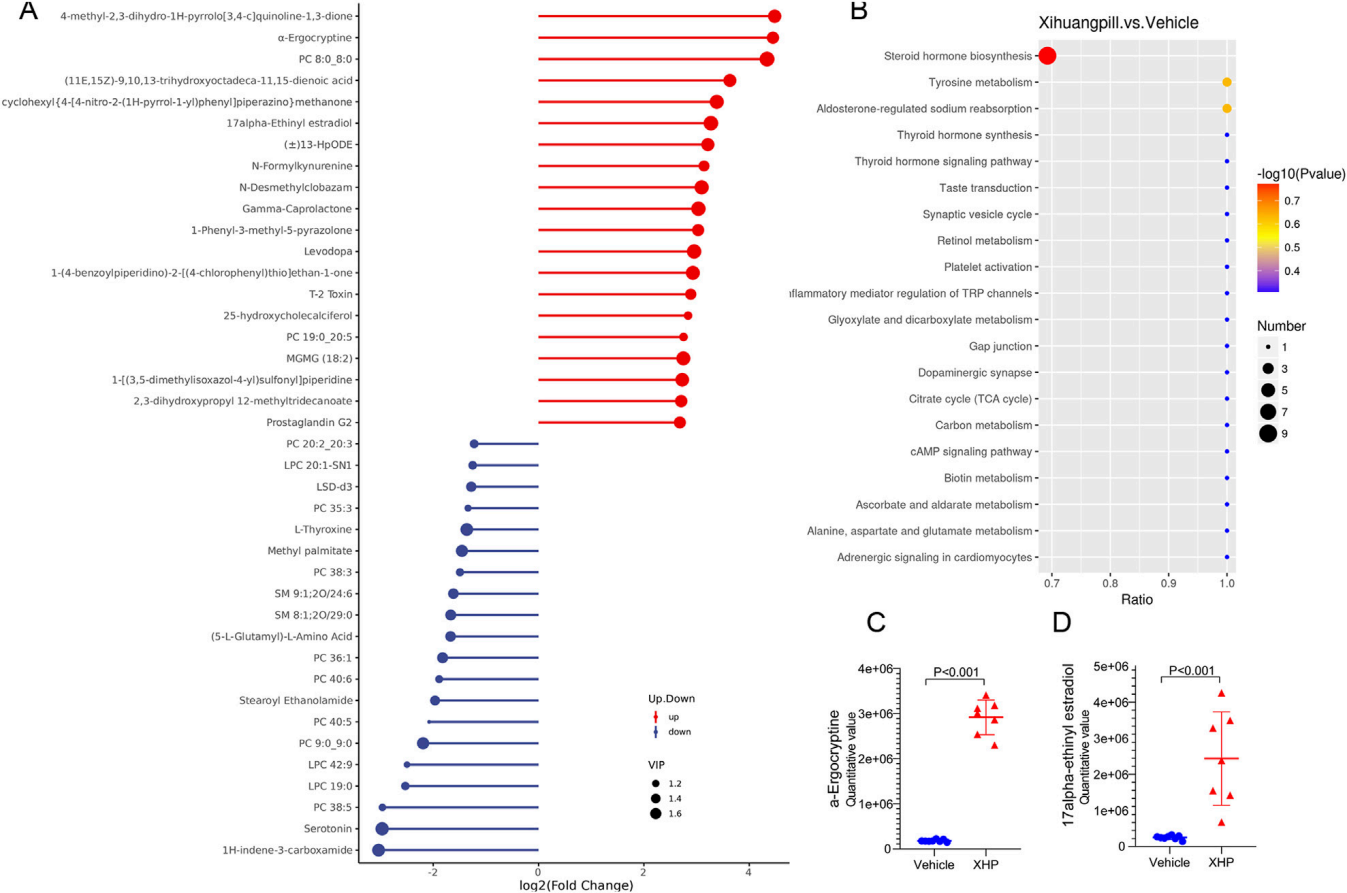
**(A)** Matchstick plots illustrating the top 20 representative metabolites that exhibit significant changes following XHP treatment. **(B)** KEGG enrichment analysis of the differential metabolites between the vehicle and XHP groups. **(C)** Quantitative analysis of the 17alpha-Ethinyl estradiol levels in both the vehicle and XHP groups. **(D)** Quantitative assessment of α-ergocryptine levels in the vehicle and XHP groups.

**FIGURE 6:**
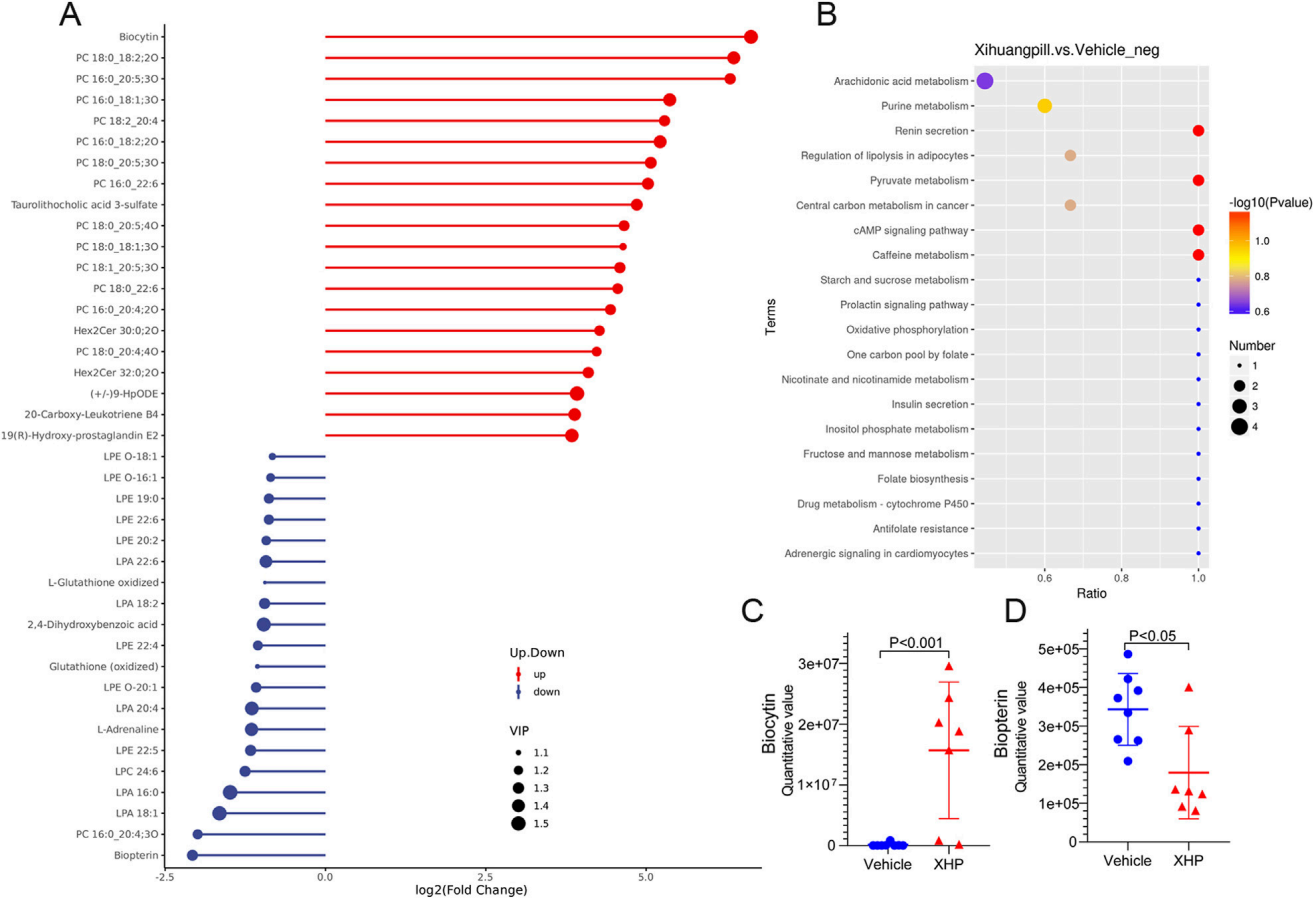
Matchstick plots illustrating the top 20 representative metabolites that exhibit significant changes following XHP treatment. **(B)** KEGG enrichment analysis of the differential metabolites between the vehicle and XHP groups. **(C)** Quantitative analysis of biocytin levels in both the vehicle and XHP groups. **(D)** Quantitative assessment of biopterin levels in the vehicle and XHP groups.

To elucidate the dynamics of metabolite alterations, heatmaps were employed to graphically represent the two sets of differential metabolites. As illustrated in Figures 3E, 4E, the heatmap utilized a color gradient, with red indicating elevated metabolite levels and blue signifying reduced levels. The heatmap analysis revealed that the samples from the vehicle and XHP groups segregated into distinct clusters, underscoring the intra-group similarity in metabolomic profiles. Complementary to the heatmaps, a matchstick plot was constructed to highlight the top 20 metabolites with the most significant changes (Figures 5A, 6A). In the ESI+, specific metabolites such as 4-methyl-2,3-dihydro-1H-pyrrolo [3,4-c] quinoline-1,3-dione, α-ergocryptine, and 17α-ethinyl estradiol exhibited substantial increases following XHP treatment. Conversely, in the ESI-, biocytin displayed the most pronounced upregulation, whereas biopterin showed the most significant downregulation relative to the vehicle group. These observations suggest that XHP intervention induces considerable shifts in the biochemical composition, particularly affecting lipid compounds, in the serum metabolome of the treated mice when compared to the control group.

### Metabolic pathway analysis

To deepen our comprehension of the metabolic processes associated with the identified metabolites, a metabolomic pathway analysis was conducted using MetaboAnalyst 5.0. This analysis involved importing the differential metabolites and screening the pathways based on the impact value derived from pathway topological analysis and the *p*-value obtained through pathway enrichment analysis. Pathways with both a high impact value and a significant *p*-value were deemed the most influential. As depicted in Figures 5B, 6B, the metabolic pathways predominantly affected by XHP included steroid hormone biosynthesis, arachidonic acid metabolism, purine metabolism, and renin secretion. Furthermore, boxplots were employed to highlight key metabolites with significant level variations. Notably, as shown in Figures 5C, D, 17α-ethinyl estradiol and α-ergocryptine exhibited the most pronounced differences in their levels between the XHP group and the vehicle group.

### Effect of XHP on the gut microbiota in CRC mice

**FIGURE 7:**
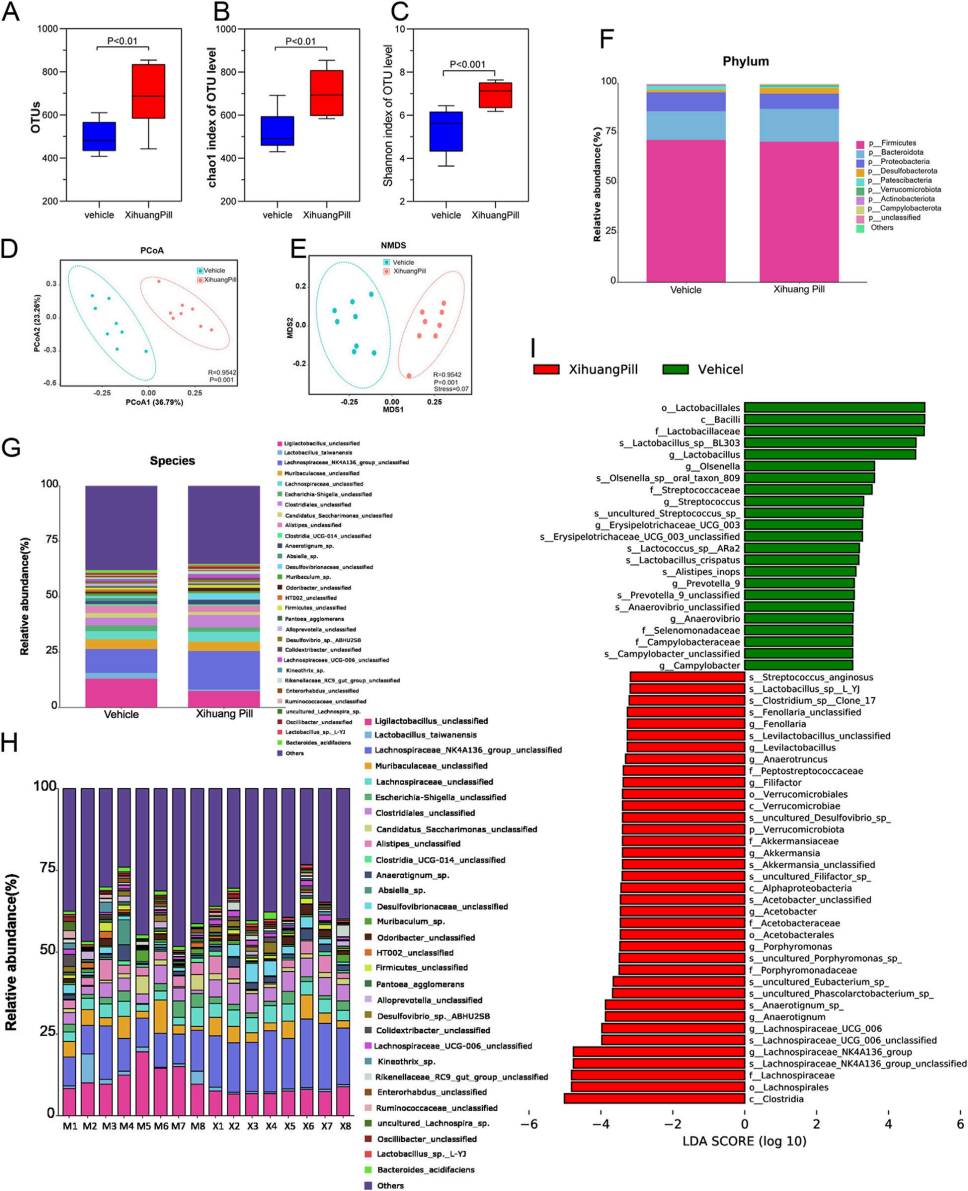
XHP administration remodels microbial dysbiosis. **(A)** Comparative analysis of observed bacterial species in the vehicle and XHP groups. **(B)** Evaluation of the Chao1 index for the vehicle and XHP groups. **(C)** Assessment of the Shannon index for the vehicle and XHP groups. **(D)** Principal Coordinate Analysis (PCoA) score plots. **(E)** Nonmetric Multidimensional Scaling (NMDS) score plots. **(F)** Analysis of bacterial taxa differences at the phylum level between the vehicle and XHP groups. **(G, H)** Analysis of bacterial taxa differences at the species level between the vehicle and XHP groups. **(I)** Analysis of bacterial taxa differences between the vehicle and XHP groups using Linear Discriminant Analysis Effect Size (LEfSe).

Given the established link between gut dysbiosis and tumorigenesis, as well as its influence on therapeutic outcomes, we conducted a 16S-rDNA gene sequencing analysis of colonic contents. This analysis aimed to ascertain whether the anti-tumorigenic effects of XHP were mediated through modulation of the gut microbiota. The alterations in microbial composition are illustrated in Figures 7A–C and Supplementary Figure S1. In terms of α-diversity, the XHP group exhibited a higher number of OTUs and Chao 1 indices compared to the vehicle group, suggesting that XHP treatment was correlated with enhanced species evenness. Furthermore, the β-diversity, as indicated by the Shannon index, was significantly distinct between the groups, implying a greater species diversity in the XHP group. The scatter plot derived from PCoA based on the unweighted UniFrac metric revealed that the two groups formed distinct clusters, indicative of a separation in microbial community structures (Figure 7D). Similar findings were observed in Non-metric Multidimensional Scaling (NMDS) analysis (Figure 7E). Altogether, these findings indicate that XHP supplementation significantly enriched the diversity and richness of the gut microbiome, potentially contributing to its anti-tumorigenic effects.

We conducted a detailed analysis of the gut microbiota composition for each sample. The gene taxonomy was annotated with reference to the SILVA database (https://www.arb-silva.de/documentation/release-138/) and the NR (RefSeq non-redundant proteins) database within the bacterial domain. Predominantly, Bacteroidetes and Firmicutes were the most abundant phyla, with a ratio of Firmicutes to Bacteroidetes (F/B ratio) of 60.34% in the vehicle group and 59.31% in the XHP group, as depicted in Figure 7F. No significant differences were observed at the phylum level between the two groups. At the species level, OTUs were carefully screened, and taxa with an average relative abundance of less than 0.1% were excluded from the analysis. In total, 57 distinct taxa (species) were identified. The average relative abundance of the top 30 bacteria is presented in Figure Figure 7G. Additionally, the distribution of taxa across each sample was visualized using a histogram, as shown in Figure 7H. Notably, XHP administration was associated with an increase in *Lachnospiraceae_NK4A136_group*, *Clostridiales*, *Desulfovibrionaceae*, and *Anaerotignum_*sp., while it led to a decrease in *Ligilactobacilus*, *Lactobacillus_taiwanensis*, and *Candidatus_saccharimonas*. Among the top 30 species, *Lachnospiraceae_NK4A136_group* exhibited the most significant change between the two groups. These findings suggest that XHP modulates the gut microbiota structure, potentially contributing to its therapeutic effects.

To further assess the differential consistency among taxa across the study groups, we employed LDA coupled with LEfSe. Taxa with an LDA score exceeding two and a *p*-value of less than 0.05 were deemed statistically significant, as previously described (Fazlollahi et al., 2018). Taxa were ranked by their effect size after being filtered through the established criteria. LEfSe results revealed that *c_Clostridia, o_Lachnospirales, f_Lachnospiraceae,* and *s_Lachnospiraceae_NK4A136_group* were the most significantly enriched in the XHP group, contributing the most to the taxa profiling. In contrast, *o_lactobacillales, c_Bacilli, f_Lactobacillaceae, s_Lactobacillus_sp_BL303* were found to be the most prominent contributors to the vehicle group’s profile, as illustrated in Figure 7I. These results underscore the distinct microbial signatures associated with XHP treatment, highlighting the potential role of specific gut microbiota in mediating the therapeutic effects of XHP.

### Correlation analysis of the gut bacteria with serum metabolites

**FIGURE 8:**
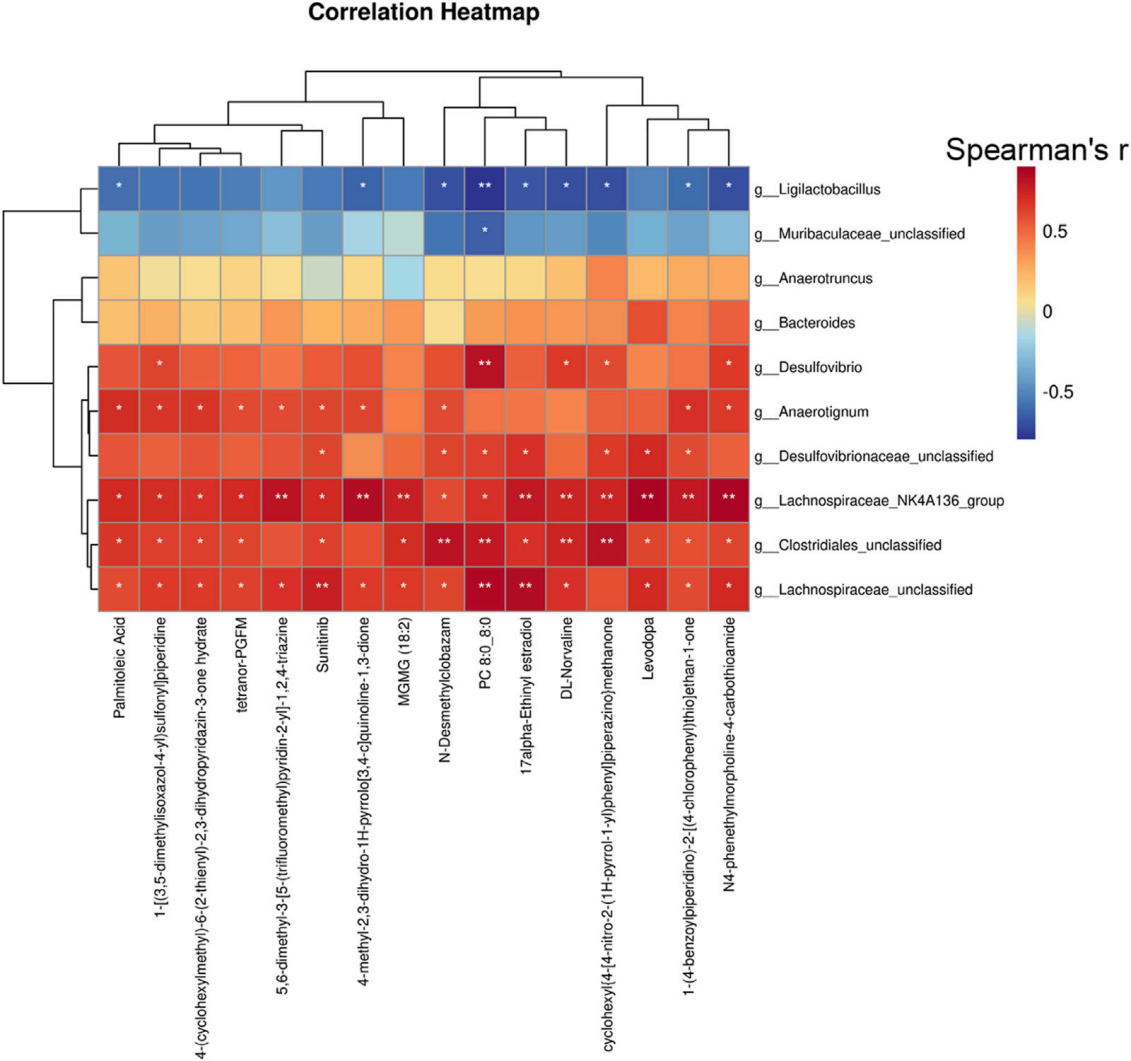
Spearman’s correlation analysis of metabolites and gut bacteria.

To gain a deeper understanding of the interplay between the biotransformation products of bacteria and the endogenous metabolic responses to XHP, we conducted a Spearman correlation analysis to explore the potential relationships between bacterial species and serum metabolites. We focused on the top 20 serum metabolites and the top 10 bacterial species based on their relative abundance and variability. The Spearman correlation heatmap at the species level revealed significant positive correlations between specific serum metabolites, including palmitoleic acid, suntinib, 17α-ethinyl estradiol, and levodopa, with certain gut bacterial taxa such as *g_Lachnospiraceae, g_Clostridiales, g_Lachnospiraceae_NK4A136_group, g_Desulfovibrionaceae,* and *g_Anaerotignum* as depicted in Figure 8. These findings suggest that XHP modulates the gut microbiota composition, which in turn may regulate the production of steroid hormones. This regulatory effect on the microbiota-steroid axis could potentially contribute to the chemopreventive activity of XHP against CRC tumorigenesis.

### Results of immunohistochemical staining

**FIGURE 9:**
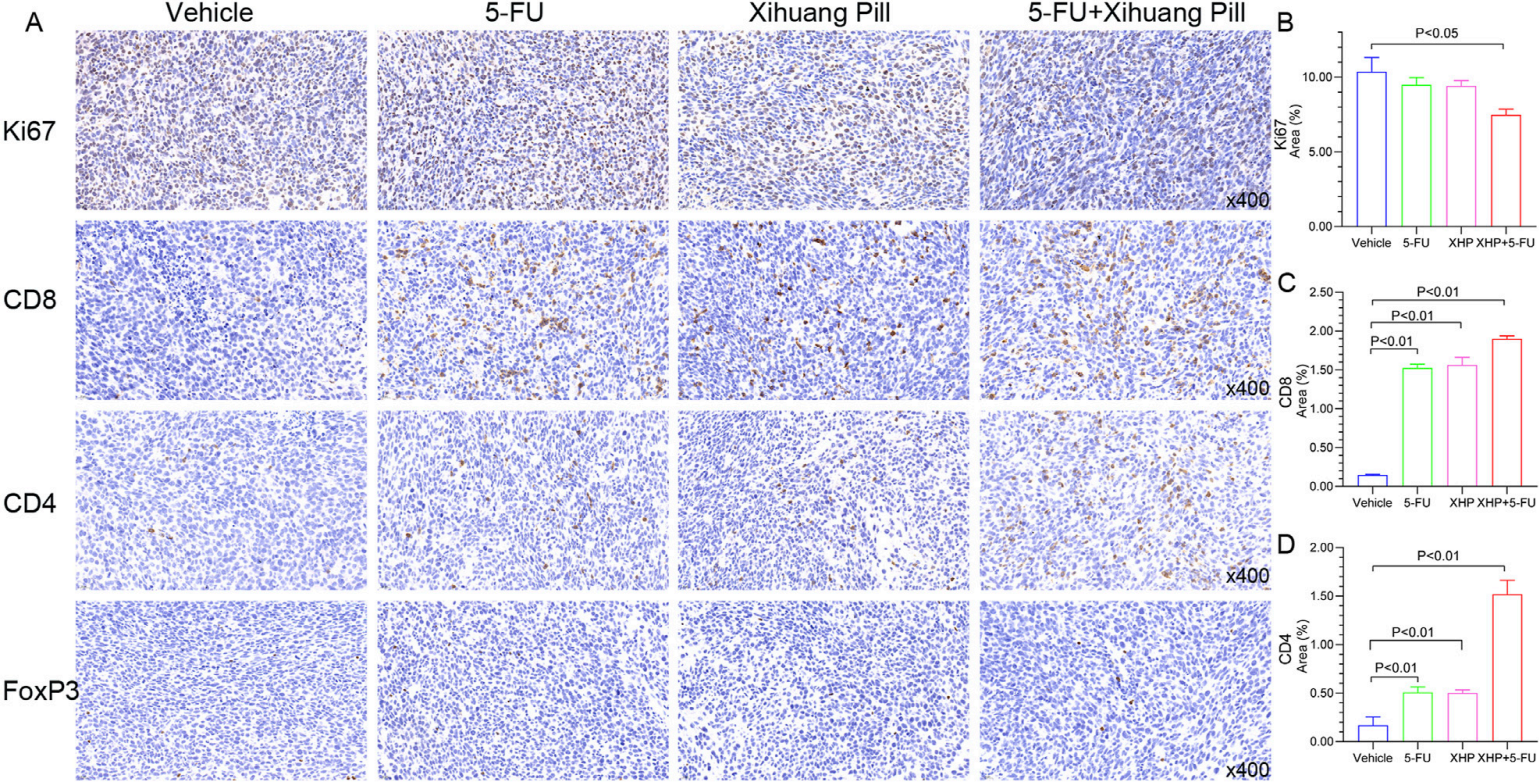
Representative photographs were obtained using the Caseviewer 2.4 imaging system. Tumor sections from all four groups, immunolabeled for Ki-67, CD8, CD4, and FoxP3, were scanned with the Caseviewer 2.4 imaging system. Semi-quantitative analyses were conducted using Image Pro Plus 6.0.

Immunohistochemical examination of tumor sections from the CT26 murine model was performed to evaluate the expression profiles of pivotal immune-related biomarkers. The illustrative staining images and the semi-quantitative analysis of these markers are depicted in Figure 9. Both 5-FU and XHP treatments resulted in a reduction of Ki67 levels, albeit not significantly different from the vehicle group. In contrast, the combined therapy markedly reduced Ki67 expression. Regarding CD8, all treatment groups exhibited an increase in CD8 expression relative to the vehicle group. Notably, the combined therapy induced a pronounced upregulation of CD4, surpassing the effects observed with 5-FU and XHP treatments. Interestingly, the expression of FoxP3, a marker for regulatory T cells, was minimal across all treatment groups. These immunohistochemical findings imply that XHP may contribute to the modulation of CD8^+^ and CD4^+^ T cell recruitment and activation, potentially enhancing the antitumor immune response. The lack of significant FoxP3 expression suggests that regulatory T cell activity may not be a predominant factor in the treatment effects observed.

## Discussion

Epidemiological research ascertains that CRC is a highly prevalent and deadly malignancy. Although immune checkpoint inhibitors have improved survival rates, the benefits for CRC patients are limited due to the scarcity of responsive subtypes. The MSI status of a tumor has profound effects on its immune microenvironment, with MSI-H CRCs typically exhibiting a higher mutational load and a more inflamed microenvironment, which can be associated with better responses to immunotherapy. The distinct immune landscapes of MSI-H and MSS tumors, characterized by varying degrees of immune cell infiltration and cytokine profiles, are of significant clinical relevance. Therefore, it is crucial to delve into the development of novel combination therapies, especially for patients with MSS tumors. The CT26 colon carcinoma cell line stands out as the most widely utilized syngeneic murine model in the field of oncology, facilitating drug development. Notably, these cells are characterized by their MSS phenotype and exhibit traits akin to the aggressive, undifferentiated, and therapy-resistant nature observed in human CRC cells (Alwarawrah et al., 2010; Castle et al., 2014; Wu et al., 2022). Thus, in this study, we have employed the CT26 tumor model as a robust testing platform for pharmacological investigations, aiming to augment the potency of existing treatment protocols.

XHP is recognized as a patented medicinal product with demonstrated efficacy in combating cancer. (Ge et al., 2022). Studies have shown that XHP can inhibit angiogenesis, potentiate the efficacy of chemotherapy, curb invasion and metastasis, and mitigate side effects both *in vivo* and *in vitro*. (Guo et al., 2015; Su et al., 2018; Wang et al., 2014; Yang et al., 2020; Zhao et al., 2020). However, the impact of XHP on CRC and the mechanisms have not been exhaustively or systematically investigated. This study aims to explore the chemopreventive effects of XHP on CRC through a comprehensive investigation integrating untargeted metabolomics and gut microbiome analysis. As anticipated, our findings indicate that XHP effectively suppressed tumor growth in the CT26 murine model, corroborating previous research (Wang et al., 2014). Furthermore, the concurrent administration of XHP with 5-FU demonstrated a synergistic therapeutic effect.

XHP is a complex formulation comprising numerous compounds, such as boswellic acid, beta-caryophyllene, and guggulsterone (Fu et al., 2020). Despite the presence of these compounds, their trace quantities pose a challenge in identifying the principal bioactive compounds of XHP. Fortunately, untargeted metabolic profiling offers a novel avenue for elucidating the key metabolites modulated during XHP treatment. In our study, we discovered that lipids and lipid-like molecules were significantly altered by XHP, aligning with previous findings (Li et al., 2020). Functional pathway analysis revealed that pathways such as steroid hormone biosynthesis, arachidonic acid (AA) metabolism, renin metabolism, pyruvate metabolism, and the cAMP signaling pathway may underlie the biological effects of XHP. Notably, steroid hormone biosynthesis, characterized by the highest fold change and statistical significance, could be a pivotal biological process influenced by XHP.

Literature reviews have established that the aforementioned pathways are intricately involved in the pathogenesis of CRC. For instance, AA has been shown to impede cell proliferation in two colon tumor models, highlighting its potential in CRC management (Oktem et al., 2023; Petry et al., 1984). The renin-angiotensin system, with its inhibitors, has garnered research interest due to their chemopreventive properties against CRC (Dierssen-Sotos et al., 2017; Dougherty et al., 2014). Pyruvate, a key glycolytic metabolite in energy metabolism, has emerged as a significant molecule following its discovery as a repressor of histone gene expression and an inhibitor of cancer cell proliferation (Ma et al., 2019). Intriguingly, the modulation of pyruvate within the tumor microenvironment is closely associated with the cytotoxicity of CD8^+^ T cells (Elia et al., 2022). The cAMP signaling pathway has been demonstrated to regulate apoptosis across various model systems, and its accumulation is known to suppress T cell receptor signaling, thereby influencing T cell functionality (Barrett et al., 2022).

Within the realm of steroid hormone biology, our study has revealed a noteworthy discovery: a pronounced upregulation of 17α-ethinyl estradiol subsequent to XHP administration. This finding offers fresh insights into the hormonal modulation induced by XHP and its potential impact on colorectal cancer. While CRC affects both sexes, a disparity in incidence rates has been documented in numerous studies (DeCosse et al., 1993). Notably, a meta-analysis has indicated that hormone replacement therapy (HRT) significantly reduces the risk of CRC (Cho et al., 2007). Accumulating evidence underscores the pivotal role of sex hormones in the pathogenesis of CRC (Jacenik et al., 2019). Indeed, this has spurred an upsurge in the exploration of natural compounds that emulate the effects of 17β-estradiol (E2) in cancer therapy, despite the mechanisms of estrogen-like actions remaining not fully elucidated (Galluzzo et al., 2008; Galluzzo and Marino, 2006; Marino, 2014). Collectively, this evidence substantiates the hypothesis that the maintenance of hormonal equilibrium may be a critical aspect of XHP’s chemopreventive strategy.

Considering the established link between microbial dysbiosis and colorectal tumorigenesis, as well as the interplay between metabolic disorders and gut microbiota perturbations (Artemev et al., 2022), our study delved into the interaction between XHP and the gut microbiota. Employing 16S-rDNA sequencing, we scrutinized the shifts in gut microbiota composition within colonic contents subsequent to XHP administration. Predictably, XHP exerted an impact on both α and β diversity. In terms of specific taxa, XHP notably enriched the *c_Clostridia, o_Lachnospirales, f_Lachnospiraceae,* and *s_ Lachnospiraceae_NK4A136_group,* with *Lachnospiraceae* recognized for its protective role in the gut, including suppression of colitis and enhancement of CD8^+^ T cell immune surveillance, thereby potentially curbing CRC progression (Chen et al., 2017; Zhang et al., 2023). Although *Clostridiales* are often perceived as detrimental (Gagniere et al., 2016), they possess the capacity to activate estrogens through deconjugation, which may have implications for CRC (Flores et al., 2012; McIntosh et al., 2012). The increase in *Desulfovibrionaceae*, typically sulfate-reducing bacteria found in soil environments (Rooney-Varga et al., 1998), could be attributed to the sulfate-containing compounds in XHP. Furthermore, the reduction in *Candidatus Saccharimonas* has been correlated with a decrease in intestinal damage (Cruz et al., 2020). In sum, XHP induced alterations in the gut microbiota structure, and its chemopreventive effects may be partially attributed to the protective, metabolic, and immunological contributions of the microbiota (Eckburg et al., 2005).

A multitude of studies have demonstrated that the gut microbiota can enhance the chemopreventive properties of natural products through the production of metabolites, interactions with the immune system, and the release of bioactive mediators. In this context, to elucidate the interplay between the microbiota and metabolites, we conducted a correlation analysis between gut microbiota composition and serum metabolite profiles. Notably, 17α-ethinyl estradiol, a key metabolite, exhibited a positive correlation with several microbial taxa, including *Lachnospiraceae*, *Clostridiales*, *Desulfovibrionaceae*, and *Anaerotignum*. 17α-ethinyl estradiol has been recognized for its protective role in CRC (Song et al., 2022), and *Lachnospiraceae* are known to modulate estrogen levels within the gut microbiome (Wang and Huycke, 2015). Furthermore, *Clostridiales* are implicated in the metabolic conversion of estrogens. These observations underscore the potential importance of steroid hormones, particularly estrogens, as a primary pathway through which XHP may exert its effects on CRC. The extent to which the chemopreventive efficacy of XHP is microbiota-dependent remains to be determined and warrants further investigation.

Beyond the recognized impacts of XHP on the modulation of microbiota and metabolic processes, our study also seeks to explore its potential influence on the micro-immune environment. The IHC analysis of CD4 and CD8 expression levels indicates that XHP may participate in lymphocytic activities. Considering the MSS subtype’s typically muted immune response (Overman et al., 2018), the strategic application of XHP could potentially enhance this restrained reaction. Our findings collectively suggest that XHP’s therapeutic modulation in CRC is likely due to its comprehensive influence on the gut microbiota, serum metabolites, and the immune microenvironment. The interplay among these systems implies that XHP might have a systemic effect, with microbiota adjustments potentially leading to altered metabolite profiles that subsequently influence immune responses. This multifaceted engagement could be pivotal to the therapeutic advantages attributed to XHP in the treatment of CRC.

Nevertheless, our research is subject to certain constraints due to the intricate nature of the experimental setup. Initially, we opted for a single-dose study rather than exploring multiple dosing strategies. This decision was pivotal in establishing the foundational efficacy and safety profile of XHP within a regulated experimental framework. It enabled us to pinpoint the initial biological reactions and to delineate potential therapeutic opportunities for XHP intervention. Furthermore, in contrast to the subcutaneous tumor model, orthotopic transplantation could offer a more authentic scenario for dissecting the intrinsic mechanisms at play. Hence, a broader experimental design, encompassing diverse dosing protocols, a range of animal models, and supplementary *in vitro* and clinical investigations, would yield a more comprehensive insight into XHP’s therapeutic capabilities. Such a strategy is indispensable for uncovering the nuances of dose-response dynamics, the chronic impacts, and the underlying mechanisms of XHP within the spectrum of CRC.

## Conclusion

In our current study, we have meticulously conducted an extensive microbiome and metabolomic analysis to affirm the therapeutic potential of XHP as a potent anti-CRC substance. It has been shown to possess the capability to serve as a valuable adjunct in the clinical handling of CRC. XHP’s influence extends to the modulation of the gut microbiota, the regulation of serum metabolite patterns, and the alteration of the tumor microenvironment. Our research findings shed light on the diverse mechanisms by which XHP operates and lay the groundwork for the development of future therapeutic approaches that harness XHP to combat CRC.

## Data Availability

The original contributions presented in the study are publicly available. This data can be found here: https://figshare.com/s/4a31f0577e4930da5f3a?file=50631171. Further inquiries can be directed to the corresponding author.
