# Supplementary figures and images for: Therapeutic potential of Xihuang Pill in colorectal cancer: Metabolomic and microbiome-driven approaches
Source: Front Pharmacol. 2024 Dec 2;15:1402448. doi: 10.3389/fphar.2024.1402448 (PMC11646767; doi:10.3389/fphar.2024.1402448)

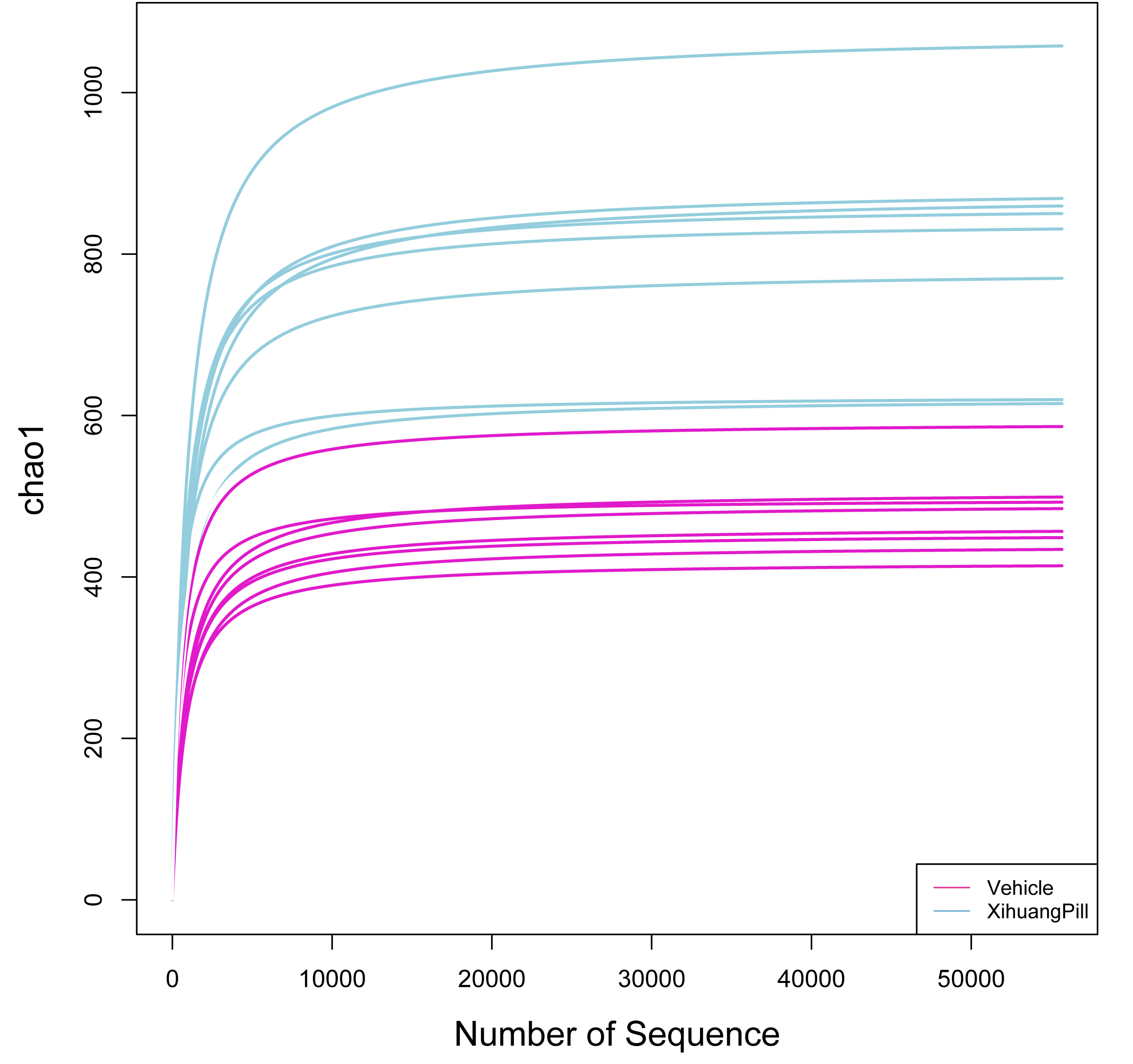

Supplement: Supplementary file 1 [file Image1.JPEG]
