# Supplementary material for: Therapeutic potential of Xihuang Pill in colorectal cancer: Metabolomic and microbiome-driven approaches
Source: Front Pharmacol. 2024 Dec 2;15:1402448. doi: 10.3389/fphar.2024.1402448 (PMC11646767; doi:10.3389/fphar.2024.1402448)
Supplement: Supplementary file 2 [file Table1.DOC]

Table S1 PCR amplification and 16S rDNA sequencing

| Region | Primers |
| --- | --- |
| V3-V4[1] | 341F(5'-CCTACGGGNGGCWGCAG-3')  805R(5'-GACTACHVGGGTATCTAATCC-3’) |
| Archae[3] | F(5'-GYGCASCAGKCGMGAAW-3')  R(5'-GGACTACHVGGGTWTCTAAT-3') |
| V4[2] | 515F(5'-GTGYCAGCMGCCGCGGTAA-3')  806R (5'- GGACTACHVGGGTWTCTAAT-3') |
| V4-V5 | F(5'-GTGCCAGCMGCCGCGG-3')  R(5'-CCGTCAATTCMTTTRAGTTT-3') |

Referrences

[1] Logue Jürg B,Stedmon Colin A,Kellerman Anne M et al. Experimental insights into the

importance of aquatic bacterial community composition to the degradation of dissolved organic

matter.ISME J, 2016, 10: 533-45.

[2] Walters W, Hyde E R, Berglyons D, et al. Improved Bacterial 16S rRNA Gene (V4 and V4-5)

and Fungal Internal Transcribed Spacer Marker Gene Primers for Microbial Community

Surveys. Msystems, 2015, 1(1):e00009-15.

[3] Takai K, Horikoshi K. Rapid detection and quantification of members of the archaeal community by quantitative PCR using fluorogenic probes. Appl Environ Microbiol. 2000 Nov;66(11):5066-72.
